# Emerging roles of hydrogel in promoting periodontal tissue regeneration and repairing bone defect

**DOI:** 10.3389/fbioe.2024.1380528

**Published:** 2024-04-24

**Authors:** Wendi Guo, Hongbin Dong, Xing Wang

**Affiliations:** ^1^ Department of Prosthodontics and Implant Dentistry, The First Affiliated Hospital of Xinjiang Medical University, Urumqi, China; ^2^ Affiliated Stomatological Hospital of Xinjiang Medical University, Urumqi, China; ^3^ Stomatology Research Institute of Xinjiang Uygur Autonomous Region, Urumqi, China

**Keywords:** hydrogel, periodontal tissue regeneration, bone tissue engineering, bone defect, repair

## Abstract

Periodontal disease is the most common type of oral disease. Periodontal bone defect is the clinical outcome of advanced periodontal disease, which seriously affects the quality of life of patients. Promoting periodontal tissue regeneration and repairing periodontal bone defects is the ultimate treatment goal for periodontal disease, but the means and methods are very limited. Hydrogels are a class of highly hydrophilic polymer networks, and their good biocompatibility has made them a popular research material in the field of oral medicine in recent years. This paper reviews the current mainstream types and characteristics of hydrogels, and summarizes the relevant basic research on hydrogels in promoting periodontal tissue regeneration and bone defect repair in recent years. The possible mechanisms of action and efficacy evaluation are discussed in depth, and the application prospects are also discussed.

## 1 Introduction

Periodontal disease is a prevalent condition that significantly impacts both dental health and overall wellbeing. Periodontal bone defects, known as a serious consequence of periodontal disease, are challenging to repair, leading to significant impacts on patients’ chewing function, aesthetics, and quality of life (Sedghi et al., 2021). Conventional periodontal bone grafting or bone substitute implantation surgery comprises the transplantation of bone or substitute materials to address alveolar bone defects resulting from periodontitis. These surgeries aim to stimulate new bone formation, repair bone defects, restore the anatomical shape of the alveolar bone, and achieve optimal periodontal tissue regeneration (Gao et al., 2024a). Nevertheless, this method is subject to numerous influencing factors and may not consistently yield satisfactory outcomes in periodontal tissue regeneration and bone defect repair. Some bone defects cannot be perfectly repaired. Hydrogels are a new type of functional polymer material that has emerged in recent years. They are cross-linked three-dimensional hydrophilic polymer networks with properties superior to traditional materials, including softness, non-deformability, strong water absorption capacity, intelligence, high drug utilization rate, safety, and convenience (Li et al., 2022a; Li et al., 2024). However, single-component hydrogels have relatively simple structures, generally low mechanical strength, and only basic hydrogel properties, which cannot fully meet the needs of complex applications and have certain limitations. In recent years, the basic research and clinical applications of hydrogels have become increasingly rich, with great potential and unique therapeutic plasticity in promoting periodontal tissue regeneration and repairing bone defects. Firstly, hydrogels can provide a microenvironment similar to the extracellular matrix, allowing periodontal tissues and bone cells to adhere, proliferate, and differentiate. Secondly, hydrogels can serve as drug release carriers, exerting anti-inflammatory and antibacterial effects, and promoting periodontal tissue regeneration. Additionally, hydrogels can also carry specific bioactive factors such as stromal cell-derived factor-1 (SDF-1) and bone morphogenetic proteins (BMPs), inducing them to differentiate into osteoblasts, thereby accelerating periodontal bone tissue regeneration. Hydrogels are currently one of the hottest research materials, and are expected to provide a new perspective for promoting periodontal tissue regeneration and repairing periodontal bone defects. Therefore, based on the latest literature on hydrogels, this article provides a review of the molecular mechanisms and efficacy evaluation of promoting periodontal tissue regeneration and repairing periodontal bone defects, and looks forward to their clinical application prospects.

## 2 Periodontal disease and bone defect

Periodontitis is a chronic inflammatory disease affecting the supporting tissues of the teeth, leading some patients to suffer from varying degrees of periodontal bone defects, significantly impacting their physical and mental wellbeing (Dannewitz et al., 2021). The treatment for periodontitis aims not only to remove causative factors and halt disease progression but also to restore damaged periodontal tissues to their original structure and function, achieving the ideal goal of periodontal tissue regeneration. The surgical treatment to promote periodontal tissue regeneration is called regenerative surgery, which mainly includes bone grafting and guided tissue regeneration, or the combination of the two can be used (Bee and Hamid, 2022). Bone grafting is among the most effective methods to repair bone defects caused by periodontitis, traditionally using materials such as autogenous bone, allograft bone, and xenogeneic bone, among others (Smeets et al., 2022). While the efficacy of the mentioned bone graft materials in bone defect repair cannot be entirely dismissed, there are also drawbacks. For instance, autogenous bone necessitates the use of the patients’ own bone, resulting in a secondary wound at the donor site, increasing the risk of postoperative complications such as infection, wound dehiscence, and increased bleeding. Additionally, postoperative unpredictable bone resorption may hinder the repair effect and prevent reaching the optimal state. Allogeneic bone carries the risk of immune rejection and requires strict donor screening and immunosuppressive therapy. Xenogeneic bone, due to species differences, has weaker biological activity and mechanical support capabilities (Ajlan et al., 2024). Thus, the clinical demand for bone repair materials with superior biological properties has grown increasingly urgent.

## 3 Overview of hydrogel

### 3.1 Basic composition of hydrogel

**FIGURE 1:**
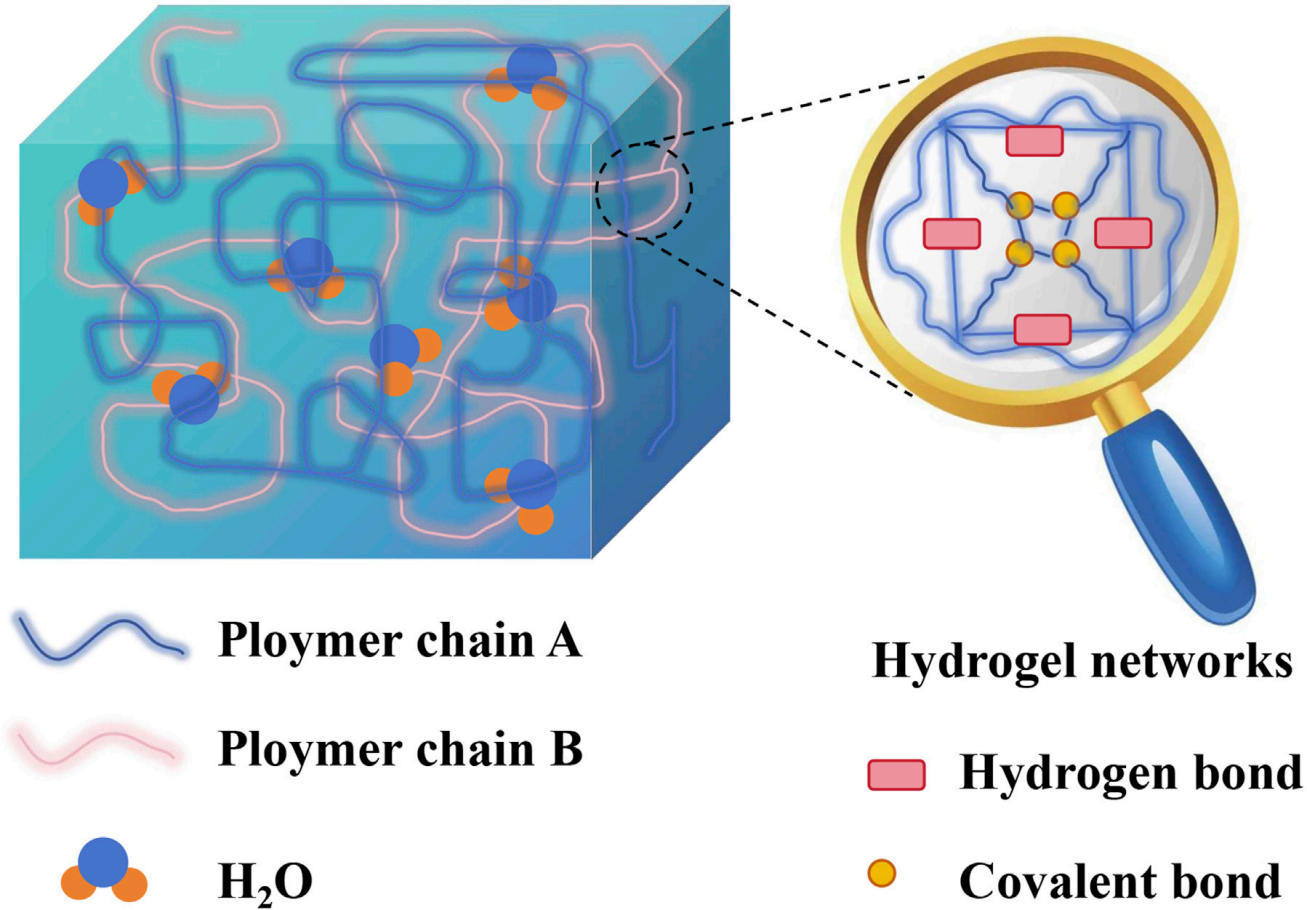
The general composition of hydrogel.

Hydrogels are three-dimensional hydrophilic polymer networks formed by the crosslinking of hydrophilic polymer chains through various interactions, such as chemical bonds, hydrogen bonds, and van der Waals forces (Hameed et al., 2024). Furthermore, the presence of hydrophilic groups in hydrogels enables rapid water absorption, high water retention capacity, and swelling without dissolution. This unique structure provides hydrogels with flexibility, enabling them to mimic the body’s tissue environment. They offer structural support to the defect site, facilitating repair of bone defects through intrinsic healing mechanisms (Li et al., 2022b; Ho et al., 2022; Zhang et al., 2023). The high water content of hydrogels, similar to the permeability of the extracellular matrix, facilitates the transport of oxygen and nutrients (Siddiqui et al., 2021). Owing to their excellent biocompatibility, biodegradability, high water content, adjustable properties, and similarities to the natural extracellular matrix, hydrogels are increasingly recognized as exceptional biomimetic tissue engineering scaffold materials. They are considered the optimal carriers for cells, bioactive factors, and controlled release drugs. It's important to note that injectable hydrogel materials, with their *in situ* cross-linking nature, can fully adapt to and restore the irregular geometric shape of the original bone defect, achieving minimally invasive repair of bone defects (Chen et al., 2022). The relatively low adhesion of hydrogels to proteins and cells ensures that hydrogels in contact with tissues do not interfere with the metabolic processes of living organisms. Additionally, tissue metabolites can easily penetrate the hydrogel (Luo et al., 2022). The scheme illustrating the general composition of hydrogel is displayed in Figure 1.

### 3.2 Basic physical and chemical properties of hydrogel

#### 3.2.1 Biocompatibility

After the material interacts with the organism, biocompatibility is primarily reflected in two aspects. The material must withstand organismic systems without rejection or damage, and the reactions generated during this process should be non-inflammatory and non-carcinogenic (Crawford et al., 2021). Biocompatibility is crucial for the successful use of hydrogel scaffolds in tissue engineering and regenerative medicine in clinical practice (Zor et al., 2019). Researchers often utilize methods such as synthetic conjugated polymers and the addition of ions to enhance tissue reconstruction and repair and address the poor biocompatibility of hydrogels (Zhang et al., 2022a).

#### 3.2.2 Biodegradability

The biodegradability of hydrogel materials is a significant advantage. The biodegradability of hydrogel involves the gradual decomposition of the material in the body through processes such as dissolution, enzymatic digestion, and cell engulfment. Upon tissue repair, the implanted material is completely replaced by the repaired tissue, without leaving any residual material during the tissue healing process (Xia et al., 2021). Ideally, hydrogel materials should exhibit controllable biodegradability, matching the growth of cells and tissue repair rate. Excessive degradation can result in a loss of mechanical integrity before complete tissue regeneration, whereas slow degradation can lead to delayed tissue healing (Fatahian et al., 2022). Therefore, determining the appropriate degradation rate is a crucial consideration in hydrogel design. Fortunately, there has been significant recent progress by researchers, including the utilization of surface modification, polymer blending, and incorporation of alkaline particles to enhance biodegradability (Gupta et al., 2024).

#### 3.2.3 Mechanical performance

The hydrogel exhibits mechanical properties at both macroscopic and microscopic levels. On the macroscopic scale, the hydrogel scaffold offers stability and volume maintenance (Belgodere et al., 2023). On the microscopic level, cells adhered to the hydrogel matrix can sense mechanical stimuli, converting them into biochemical signals to regulate crucial physiological processes (Li et al., 2022c). Hydrogels offer extensive application potential in bone tissue engineering. Enhancing the mechanical performance of hydrogels is crucial for improving the effectiveness of bone defect repair (Zhao et al., 2022). Inadequate mechanical properties of the hydrogel material can cause the repaired defect to deform easily and fail to offer early-stage support. In order to improve the mechanical performance of hydrogels, researchers have employed diverse methods to enhance the mechanical performance of hydrogels, such as constructing double network structures (Guo et al., 2021), utilizing composite nano-technology (Cui et al., 2019), introducing conductive materials (Arambula-Maldonado et al., 2023), and reinforcing fiber networks (Brusentsev et al., 2023). Studies have demonstrated that biomaterials with mechanical properties matching those of bone can stimulate bone cell proliferation and mineralization, and effectively facilitate bone growth.

## 4 Classification of polymer-based hydrogels

**FIGURE 2:**
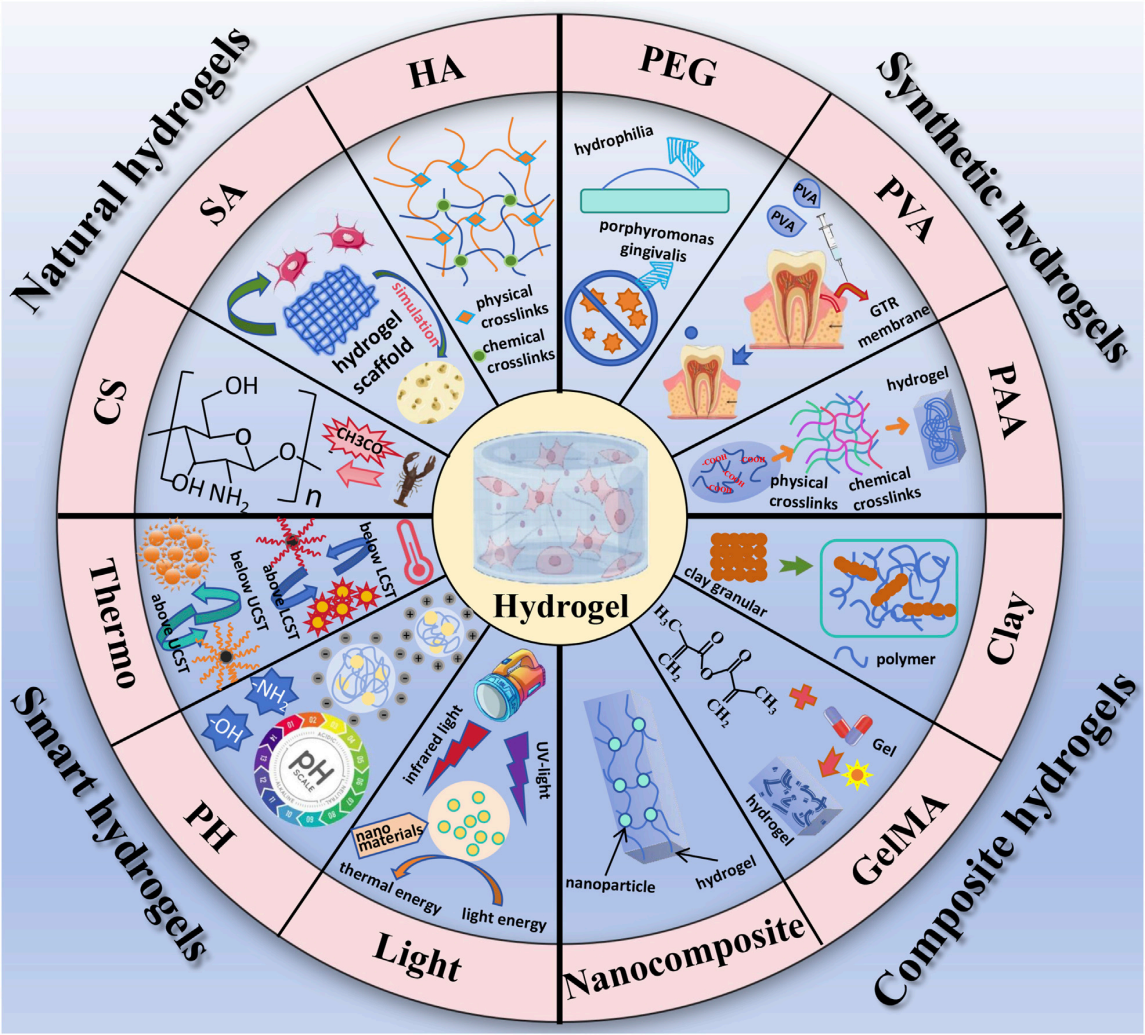
The main classification of hydolgel.

Polymer-based hydrogels can be classified into two main categories based on their source of materials (Ur Rehman et al., 2020). Naturally derived polymer-based hydrogels are primarily sourced from animals, plants, and microorganisms, exhibiting excellent biocompatibility and biodegradability. Examples include chitosan, sodium alginate, and hyaluronic acid (Sun et al., 2021). Synthetic polymer-based hydrogels are predominantly derived from common chemical raw materials, offering not only good biocompatibility but also enhanced mechanical properties. Common materials used in the preparation of synthetic polymer-based hydrogels include polyethylene glycol (PEG), polyvinyl alcohol (PVA) and polyacrylic acid (PAA). Based on their response to external stimuli, hydrogels can also be classified as traditional hydrogels and responsive hydrogels, the latter also known as intelligent responsive hydrogels. Intelligent responsive hydrogels exhibit reversible responses to environmental stimuli, with their shape, mechanical, optical, permeation rate, and recognition properties showing acute responses to changes in the surrounding microenvironment (such as temperature, pH, light, and magnetic fields), and reversible changes with the stimulating factors. It is worth mentioning that composite hydrogels, represented by clay hydrogels, have emerged as a new type of hydrogel in recent years. This composite system enhances overall mechanical properties, biocompatibility, and processability through the complementary advantages of different materials. We briefly introduce some representative hydrogel systems and their applications as followed. The main classification of hydolgel is summarized in Figure 2.

### 4.1 Natural polymer-based hydrogels

#### 4.1.1 Chitosan-based hydrogel

Chitin is usually extracted from natural shells of shrimp, crabs and insects, and part of the acetyl group is removed to obtain chitosan. Chitosan is the sole naturally occurring alkaline polysaccharide that contains free amino groups (Atia et al., 2022). Chitosan-based materials have the capacity to form chemical and physical cross-linked hydrogels through processes like exposure to ultraviolet light, pH alteration, and temperature adjustment (Yu et al., 2016). Its primary characteristic is the capacity to introduce specific functional groups with reactive properties, yielding a range of chitosan derivatives via chemical modification. Chitosan-based hydrogel systems exhibit outstanding biocompatibility, degradability, anti-inflammatory effects, broad-spectrum antibacterial properties, as well as the ability to facilitate cell adhesion, proliferation, and differentiation (Ma et al., 2022). Gu et al. (2024) used forty-eight male Wistar rats to establish a periodontitis model to detect the effects of chitosan/β-sodium glyceropylate (β-GP)/glycolic acid (GA) hydrogel carrying erythropoietin and FK506 (EPO-FK506-CS/β-GP/GA). The results showed that the hydrogel had drug stability and slow release, could significantly enhance new bone formation in the bone defect area, and had obvious bone induction properties. EPO-FK506-CS/β-GP/GA hydrogel could promote periodontal tissue regeneration. Eshwar et al. (2023) investigated an alginate-chitosan hydrogel, and clinical randomized trials indicated that the hydrogel enhanced the clinical attachment levels and fostered human bone regeneration, thus augmenting its potential in the field of tissue engineering.

#### 4.1.2 Sodium alginate-based hydrogel

Derived from marine algae cell walls and intercellular spaces, sodium alginate is a linear natural anionic polysaccharide (Liu et al., 2023). It is a natural high molecular weight polymer created by linking β-1, 4-D-mannuronic acid (M segment) and α-1, 4-L-guluronic acid (G segment) via (α-1, 4) glycosidic bonds. Due to its non-toxicity, low cost, good biocompatibility, low immunogenicity, and its capability to form gels through crosslinking with divalent or multivalent cations, it has extensive potential applications in oral tissue engineering (Sanchez-Ballester et al., 2021). The three-dimensional network structure of sodium alginate hydrogel offers a novel environment for cell adhesion and proliferation, mimicking the microenvironment of human bone tissue. Furthermore, sodium alginate hydrogel holds significant promise as a scaffold material for bone tissue engineering. It not only offers suitable physical support and biocompatibility but also guides cell proliferation to form specific tissues (Suo et al., 2023). Presently, researchers have started blending sodium alginate with other high molecular weight materials to facilitate cell growth (Ouyang et al., 2024). This blending technique offers a novel approach to develop biomedical materials with outstanding performance and is anticipated to be pivotal in the realm of tissue engineering.

#### 4.1.3 Hyaluronic acid-based hydrogel

One of the main components of the extracellular matrix is hyaluronic acid (HA), which is synthesized into HA hydrogels through physical or chemical crosslinking methods. It has good biocompatibility, does not easily cause immune reactions, and the HA hydrogel has good degradability, gradually degrading into metabolites that exist in the body without burdening the human body (Marinho et al., 2021). HA hydrogels exhibit good biocompatibility and biological activity, which have made them widely used in various fields. In bone tissue repair, HA hydrogels can provide a scaffold structure similar to the bone microenvironment to promote the differentiation of stem cells into bone cells, and promote bone regeneration through the release of growth factors and other bioactive substances (Wang et al., 2021). In tissue engineering, HA hydrogels can serve as cell carriers, providing a suitable environment to support cell adhesion, proliferation, and differentiation, thus achieving tissue regeneration and repair (Fujioka-Kobayashi et al., 2016). Despite the advantages of hyaluronic acid hydrogels including non-toxicity, biodegradability, and biocompatibility, their extremely high water absorption and enzyme degradation make them prone to corrosion and degradation in the body. Thus, combining hyaluronic acid with other smart hydrogels can yield new, environmentally friendly materials with enhanced performance (Liu et al., 2022a). The classification of natural polymer-based hydrogels is summarized in Table 1.

**TABLE 1:** Classification of natural polymer-based hydrogels.

Classification	Hydrogel matrix	Research and application	Reference
Chitosan (CS)	Chitosan/quaternized CS/nano-hydroxyapatite	This hydrogel demonstrates excellent biocompatibility and antibacterial properties, and can be utilized for the treatment of bone defects	Tian et al. (2024)
Alginate (Alg)	Alginate/gelatin/freeze-dried bone allograft nanoparticles	The hydrogel enhances cell adhesion, proliferation, and osteogenic differentiation, exhibiting significant potential to augment bone regeneration	Bastami et al. (2024)
Hyaluronic acid (HA)	Fluorenylmethyloxycarbonyl-diphenylalanine (FmocFF)/HA	The fmoff/HA hydrogel is used for acellular, biomimetic, and immunomodulatory bone tissue engineering scaffolds	Halperin-Sternfeld et al. (2023)
Cellulose	Carboxymethyl cellulose-methacrylate/hydroxyapatite	This hydrogel effectively promotes cell proliferation, supports adhesion, upregulates the expression of osteogenesis-related genes, and enhances bone regeneration, thereby increasing the strength of newly formed bone	Qiu et al. (2024)
Gelatin	Zeolitic imidazolate framework-8 (ZIF-8)/gelatin methacryloyl (GelMA)	The hydrogel promotes osteogenic differentiation of bone marrow mesenchymal stem cells and facilitates the regeneration of alveolar bone	Liu et al. (2022b)
Lignin	Lignin-copper sulfide/polyvinyl alcohol	The hydrogel exhibits high-efficiency antimicrobial and anti-biofilm activities, making it suitable for application in wound healing	Xie et al. (2022)
Silk fibroin	MXene nanosheets/regenerated silk fibroin (MXene/RSF)	The MXene/RSF hydrogel can modulate the immune microenvironment and generate new blood vessels, providing a novel strategy for bone regeneration and repair	Hu et al. (2022)

### 4.2 Synthetic polymer-based hydrogels

#### 4.2.1 Polyethylene glycol-based hydrogels

Polyethylene glycol (PEG) is a linear, neutral polyether polymer that possesses excellent biocompatibility, biodegradability, low immunogenicity, and affordability, making it an important biomaterial in the field of biomedicine. Merrill et al. (1982) first investigated PEG and polyethylene oxide (PEO) as hydrophilic biomaterials, and the results showed that the adsorption of proteins on glass surfaces could be effectively inhibited by the presence of PEO. Since then, various types of PEG have been utilized for different purposes, such as protein surface modification to confer resistance and enhance surface biocompatibility. Fraser and Benoit (2022) found that the presentation of RGD and GFOGER peptides in PEG hydrogels enhanced the functionality of periodontal ligament cells (PDLCs), and this hydrogel system effectively controlled the function and activity of PDLCs, promoting periodontal tissue regeneration. Liu et al. (2021a) studied an intelligent gingival protease-responsive hydrogel loaded with SDF-1 (PEGPD@SDF-1) and observed that the PEGPD@SDF-1 hydrogel exhibited good biocompatibility, promoting the proliferation, migration, and differentiation of periodontal ligament stem cells. Moreover, this hydrogel inhibited the proliferation of porphyromonas gingivalis, creating a low-inflammatory environment and inducing osteogenesis, thus possessing the ability to promote *in situ* regeneration of periodontal tissues.

#### 4.2.2 Polyvinyl alcohol-based hydrogel

Polyvinyl alcohol-based hydrogels, after crosslinking and swelling, form three-dimensional network-like colloidal dispersion. They possess high water absorption, degradability, good biocompatibility, and mechanical properties, making them widely applied in various medical fields (Rivera-Hernández et al., 2021). Due to the relatively simple structure and limited functionality of PVA, reinforcing components and functional materials are often incorporated into PVA hydrogel networks to improve and modify the overall performance of the hydrogel. Xiang et al. (2022) prepared a polyvinyl alcohol/hydroxyapatite/tannic acid (PVA/HA/TA) composite hydrogel, which exhibited high water content, porous structure, and good mechanical properties. *In vitro* cell experiments demonstrated excellent cell compatibility of the PVA/HA/TA composite hydrogel, promoting cell growth and adhesion, making it a promising material for bone tissue engineering. Zhou et al. (2020) developed a novel guided tissue regeneration (GTR) membrane using a composite hydrogel of polyvinyl alcohol (PVA) and fish collagen (Col). By adjusting the ratio of PVA/Col, they achieved control over the adhesion and proliferation of human periodontal ligament fibroblasts (HPDLFs) and human gingival fibroblasts (HGFs). The PVA/Col composite hydrogel exhibited unlimited potential as a GTR membrane for guiding periodontal tissue regeneration.

#### 4.2.3 Polyacrylic acid-based hydrogel

PAA is a type of synthetic polymer with high hydrophilicity and a large number of carboxyl groups. It can form hydrogels through physical or chemical crosslinking. Wen et al. (2023) prepared a novel composite mineral matrix PAA-CMC-TDM hydrogel using amorphous calcium phosphate (ACPs), PAA, carboxymethyl chitosan (CMC), and dentin matrix (TDM) as the matrix. The hydrogel exhibited good biocompatibility and degradability, and its mechanical properties could be adjusted without affecting the functional activity of TDM. The experimental results showed that the hydrogel significantly improved the differentiation ability of mesenchymal stem cells into tooth or bone, and could repair irregular hard tissue defects *in situ*. Fan et al. (2023) combined PAA and sodium alginate (SA) to obtain a double polymer network hydrogel, in which ion crosslinking and SiO2 nanoparticles were introduced as dual reinforcement materials. Compared with standard PAA hydrogel, the hydrogel exhibited enhanced adhesion and shape memory properties, and further improved biocompatibility and osteogenic potential. SA-PAA-SiO2 has great potential in bone tissue engineering. The classification of synthetic polymer-based hydrogels is summarized in Table 2.

**TABLE 2:** Classification of synthetic polymer-based hydrogels.

Classification	Hydrogel matrix	Research and application	Reference
Polyethylene glycol (PEG)	Mineral-coated microparticle/bone morphogenetic protein-2/chitosan/polyethylene glycol	This hydrogel can sustainably release growth factors and accelerate bone formation by promoting the activity of bone marrow mesenchymal stem cells	Xu et al. (2023)
Polyvinyl alcohol (PVA)	Polyvinyl alcohol/sodium alginate	PVA/SA hydrogel effectively promotes osteogenic differentiation of cells and is applied in bone tissue engineering	Zhang et al. (2024)
Polyacrylic acid (PAA)	Polyethyleneimine (PEI)/PAA-hydroxyapatite (HA)-Vancomycin (VAN)	The PEI/PAA-HA-VAN hydrogel exhibits effective antibacterial properties and promotes the expression of osteogenic genes	Wang et al. (2022)
Polyacrylamide (PAAM)	Polyurethane (PU)/Polyacrylamide/gelatin (Gel)	The PU/PAAM/Gel hydrogel can stimulate the reconstruction and growth of new bone tissue, exhibiting good osteogenic performance	He et al. (2021)
Polymethyl methacrylate (PMMA)	Gelatin-methacryloyl/polymethyl methacrylate/polydopamine (GelMA/PMMA/PDA)	The GelMA/PMMA/PDA hydrogel possesses excellent osteogenic capabilities, offering a new perspective for the treatment of bone defects	Wu et al. (2022)

### 4.3 Smart hydrogels

#### 4.3.1 Temperature-responsive hydrogels

Temperature-responsive hydrogels, also known as thermoresponsive hydrogels or temperature-sensitive hydrogels, are hydrogel materials with both hydrophilic and hydrophobic groups on their polymer chains. They exhibit temperature-responsive phase transition properties. When the temperature reaches a certain critical point, the affinity of the thermoresponsive hydrogel towards the solvent changes, leading to a swelling-shrinking transition. This temperature transition point is referred to as the lowest critical solution temperature (LCST) or utmost critical solution temperature (UCST). Injecting thermoresponsive hydrogels into periodontal pockets, they undergo an *in situ* sol-gel transition response under the stimulation of oral temperature. Xu et al. (2019) developed a thermosensitive hydrogel composed of chitosan (CS), sodium glycerophosphate (β-GP), and gelatin, which can sustainably release aspirin and erythropoietin (EPO) when injected. The study demonstrated that CS/β-GP/gelatin hydrogel is a novel drug carrier with easy preparation and excellent biocompatibility. The loaded aspirin/EPO in CS/β-GP/gelatin hydrogel exhibited significant anti-inflammatory and periodontal tissue regeneration-promoting effects. This hydrogel holds promise as a potential candidate for the clinical treatment of periodontitis.

#### 4.3.2 pH-responsive hydrogels

pH-responsive hydrogels are a type of hydrogel material that undergoes swelling or shrinking in response to changes in pH. These hydrogels contain specific acidic groups (such as carboxyl groups) or basic groups (such as amino groups). The physicochemical properties of pH-responsive hydrogels largely depend on the charge changes within the material at different pH values and the interactions between charges. Yu et al. (2022) developed a dual-crosslinked gel system with a polyhedral oligomeric silsesquioxane (POSS) matrix, surrounded by a shell of six dithiol-linked PEG and two 2-ureido-4[1H]-pyrimidinone (UPy) groups. The thiol-disulfide exchange reaction exhibited pH-responsive “on/off” functionality, allowing for controlled structure of the hydrogel. The results showed that the hydrogel improved mechanical strength and had a positive effect on the proliferation, adhesion, and osteogenic ability of periodontal ligament stem cells (PDLSCs). In summary, pH-responsive hydrogels hold great potential for various applications, including drug delivery, tissue engineering, and biomedical devices, due to their ability to respond to changes in pH and provide controlled release and targeted therapy.

#### 4.3.3 Light-responsive hydrogels

Light-responsive hydrogels are a type of hydrogel that undergo changes in their morphology, crosslinking density, and other properties under different light conditions, such as ultraviolet light, near-infrared light, or visible light. There are two different response mechanisms for light-responsive hydrogels: firstly, due to the presence of photosensitive functional groups (such as spiropyran, azobenzene, and other groups) within the hydrogel itself, the properties of the hydrogel change when it absorbs a certain amount of photon energy; secondly, by introducing nanomaterials with photothermal effects (such as gold nanoparticles, graphene oxide, etc.) into thermosensitive hydrogels, the light energy of the nanomaterials is converted into heat energy under light conditions using the photothermal effect, raising the temperature of the hydrogel and thus regulating its properties (Liu et al., 2021b). Zhai et al. (2021) studied a clay-based nanocomposite hydrogel using 4-acryloylmorpholine as a monomer. After exposure to ultraviolet light, the hydrogel exhibited good biocompatibility and mechanical properties. Additionally, animal experimental results demonstrated that the hydrogel had the ability to promote osteoblast differentiation, providing a new clinical approach for bone defect repair. Magalhães et al. (2022) prepared a nanocomposite hydrogel using laponite and polyethylene glycol diacrylate (PEGDA) and utilized ultraviolet radiation to enhance its bone regeneration ability, showcasing its potential application value in the field of bone regeneration. The classification of smart hydrogels is summarized in Table 3.

**TABLE 3:** Classification of smart hydrogels.

Classification	Stimulus condition	Control mechanism	Research and application	Reference
Thermo-responsive	Temperature	By regulating the temperature to control hydrogen bonding within the hydrogel, a sol-gel phase transition is achieved	The smart thermosensitive hydrogel possesses gelation properties and the ability to induce angiogenesis, offering a therapeutic approach for the treatment of bone defects	Lv et al. (2023)
pH-responsive	pH	Changes in pH lead to the ionization of acidic and basic groups, thereby altering the charge density of the hydrogel	The hydrogel demonstrates potent therapeutic efficacy in the treatment of periodontitis, restoration of local immune function, and eradication of pathogens	Yan et al. (2022)
Light-responsive	Light	Light-responsive hydrogels contain photosensitive molecules or groups capable of absorbing light of specific wavelengths	The hydrogel exhibits significant antibacterial properties and can promote cell adhesion and proliferation	Chang et al. (2022)
ROS-responsive	Reactive oxygen species (ROS)	After the reaction of reactive oxygen species with sensitive functional groups in the hydrogel, it will cause the fracture or crosslinking of the hydrogel network structure	ROS-respontive hydrogels can effectively alleviate inflammatory responses in periodontal tissues and reduce bone loss	Gan et al. (2023)
Enzyme-responsive	Matrix metalloproteinase 8 (MMP-8)	Based on the specific functional groups in the hydrogel reacting with specific enzymes, this leads to changes in the hydrogel structure	The MMP-8 hydrogel can inhibit the growth of Porphyromonas gingivalis and maintain its biological activity	Guo et al. (2019)
Glucose-responsive	Glucose	When glucose interacts with these specific functional groups or enzymes, it triggers a chemical or enzymatic reaction, leading to changes in the hydrogel’s structure	The hydrogels inhibit the growth of Porphyromonas gingivalis and exhibit strong antibacterial and anti-inflammatory activity	Liu et al. (2022c)

### 4.4 Composite hydrogels

Composite hydrogels refer to the incorporation of one or several polymers into a composition consisting of two or more different types or components of polymers, forming a composite system with specific structure and functionality through hydrogen bonding and electrostatic interactions. Clay hydrogels have been representative examples of composite hydrogels in recent years. Dong et al. (2021) uniformly mixed laponite nanoclay with methacrylic acid gelatin to obtain a composite hydrogel. The addition of nanoclay improved the rheological properties, degradation stability, and mechanical strength of the hydrogel. This composite hydrogel scaffold exhibited high proliferation and osteogenic differentiation capacity, making it a promising candidate for bone tissue regeneration biomaterials. Hakimi et al. (2023) developed a novel organic-mineral nanofiber hydrogel composed of chitosan-polyethylene oxide (CS-PEO)/nanoclay-alginate (NC-ALG). The inclusion of NC particles in the hydrogel improved its biocompatibility and promoted bone tissue regeneration. Zhang et al. (2022b) designed a chitosan/polyaniline/lithium polysaccharide (COL) hydrogel, in which aluminum silicate clay material laponite (LAP) was incorporated. This composite hydrogel exhibited good biocompatibility and degradability. Furthermore, the LAP-loaded composite hydrogel demonstrated excellent osteogenic differentiation capacity and could be used for bone defect repair.

## 5 Application value of hydrogel in promoting periodontal tissue regeneration

**FIGURE 3:**
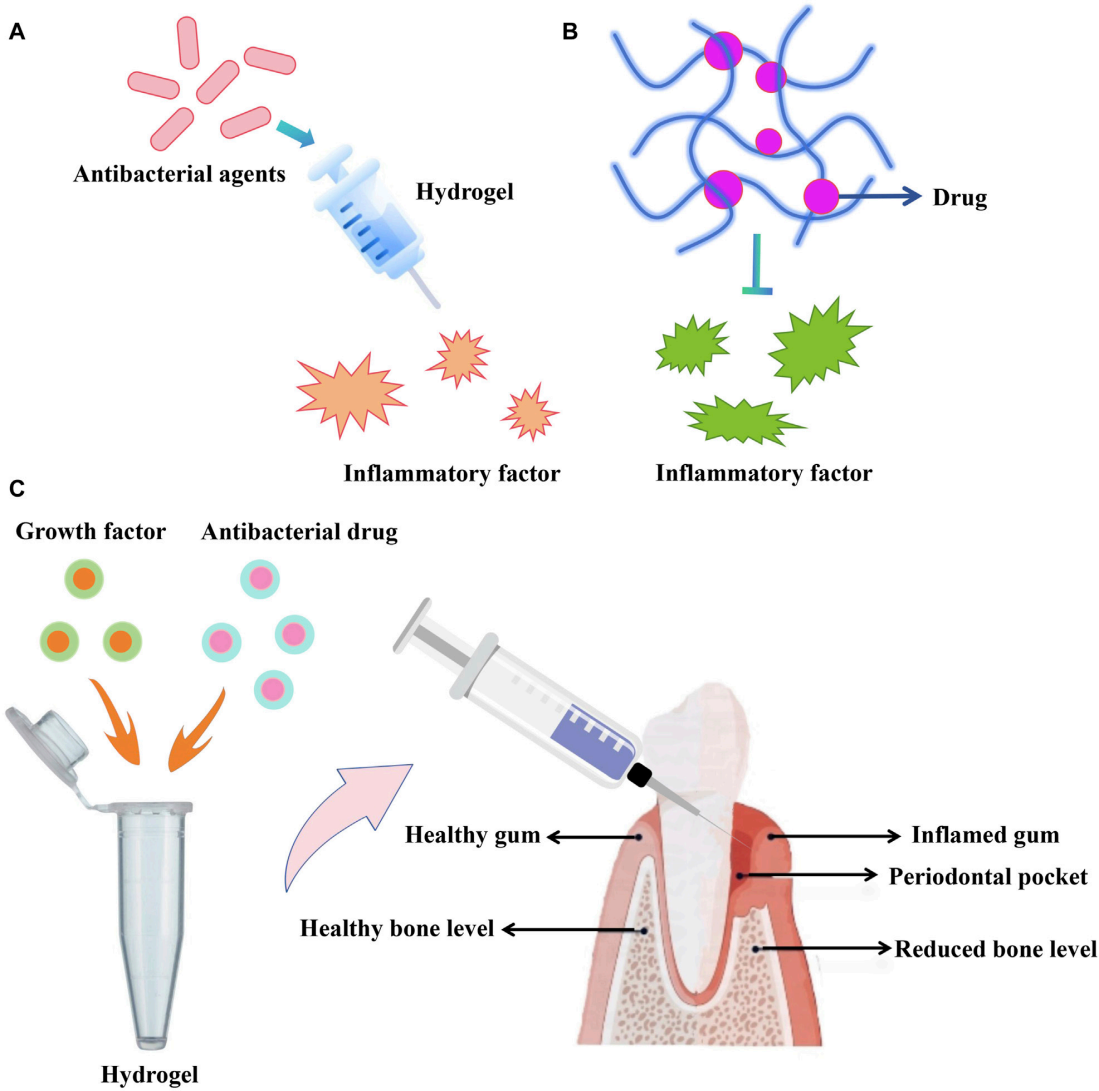
**(A,B)** Antibacterial agents or drugs can be integrated with hydrogel to suppress inflammatory factor. **(C)** Growth factor-loaded hydrogel to promote periodontal tissue regeneration.

The application research of hydrogels in periodontal tissue regeneration has continuously achieved breakthroughs. Early studies mainly focused on the antibacterial activity of hydrogels themselves, aiming to inhibit the development of infection and inflammation by adding antibacterial agents (Chen et al., 2016). However, with further research, it has been found that the effect of a single antibacterial agent is limited. To overcome these problems, researchers have begun to explore the use of hydrogels as carriers for loading different antibacterial drugs. This loading system can provide a more long-lasting antibacterial effect while reducing the amount of drugs used (Wang et al., 2020). In recent years, hydrogels can be used as carriers for carrying and releasing bioactive molecules, such as growth factors and anti-inflammatory factors. These bioactive molecules can promote tissue regeneration and repair, accelerating the healing process of periodontal tissues (Kuroda et al., 2019). Some studies have also focused on loading growth factors and antibacterial drugs together in hydrogels. This dual-functional hydrogel can not only control infection and inflammation, but also promote the regeneration and repair of periodontal tissues. The potential mechanisms employed by hydrolgel in promoting periodontal tissue regeneration are demonstrated in Figure 3. Tan et al. (2023) prepared a chitosan-based thermosensitive hydrogel loaded with β-tricalcium phosphate, which confirmed the three-dimensional network structure of the hydrogel and demonstrated significant biocompatibility with pre-osteoblastic cells MC3T3-E1 and human gingival fibroblasts, showing great potential in periodontal tissue regeneration. Ammar et al. (2018) incorporated freeze-dried platelet concentrate (FDPC) into chitosan/β-glycerophosphate lipid hydrogel, both showing sustained release of transforming growth factor and platelet-derived growth factors, significantly improving the viability of periodontal ligament stem cells, conducive to periodontal tissue regeneration, and providing essential growth factors and progenitor cells for periodontal tissue regeneration. Fawzy El-Sayed et al. (2015) used gingival-margin-derived stem/progenitor cells (G-MSCs) and hyaluronic acid-synthesized extracellular matrix (HA-sECM) hydrogel loaded with IL-1ra to explore its potential for periodontal regeneration. The results showed that this hydrogel significantly improved periodontal attachment levels, connective tissue adhesion, and alveolar bone regeneration, demonstrating significant periodontal tissue regeneration capability when used in combination with G-MSCs and IL-1-loaded HA-sECM hydrogel. Momose et al. (2016) evaluated the use of collagen hydrogel combined with fibroblast growth factor-2 (FGF2) for healing periodontal defects in beagle dogs. The results showed that periodontal tissues, periodontal ligament-like tissues, and Sharpey’s fibers in beagle dogs were repaired, and FGF2-loaded collagen hydrogel guided periodontal regeneration, restoring the function of periodontal tissues.

Currently, hydrogels have enormous potential in the treatment of periodontal tissue regeneration. However, most related research is still at the stage of animal experiments, requiring further exploration of its clinical efficacy and translational applications. With in-depth research on hydrogels and continuous technological improvement, it is believed that their application in the field of periodontal tissue regeneration will be further expanded. We can look forward to hydrogels providing new solutions for the treatment of periodontal tissue diseases, bringing more benefits to periodontal health.

## 6 Bone defect repair strategy based on hydrogel

Part of periodontitis patients have bone defects, promoting bone regeneration in the area to restore normal chewing function is particularly important for patients with periodontitis combined with bone defects (Li et al., 2023). The repair and regeneration of bone defects is a challenging and highly demanding research.

### 6.1 Hydrogels act as carrier to promote bone defect repair

Researchers are constantly exploring new methods to improve the effectiveness of bone defect repair. Hydrogels can encapsulate cells and/or growth factors, effectively protecting them from external environmental influences and maintaining their biological activity. When the hydrogel comes into contact with the site of the bone defect, it selectively transports the encapsulated cells and/or growth factors, thus promoting the proliferation and differentiation of bone cells, accelerating the regeneration and repair of bone tissue (Santos et al., 2023). In addition, hydrogels can also regulate the release rate of cells and growth factors, achieving sustained and controlled release, further enhancing the repair effectiveness (Arpornmaeklong et al., 2021). Tan et al. (2019) prepared a supermolecular hydrogel assembly of NapFFY with SDF-1 and BMP-2 for the treatment of periodontal bone defects in rats, with a bone regeneration rate as high as 56.7%. Both *in vitro* and *in vivo* results indicated that these two bioactive factors were released synchronously and continuously from the hydrogel under ideal conditions, effectively promoting the regeneration and reconstruction of periodontal bone tissue. It is expected that the SDF-1/BMP-2/NapFFY hydrogel may soon replace clinical bone transplantation for the repair of periodontal bone defects. Parisi et al. (2020) introduced adaptors of fibronectin into hyaluronic acid/polyethylene glycol-based hydrogel, evaluating its ability to promote osteogenesis in rats. The hydrogel was found to support osteoblast adhesion, accelerate platelet aggregation and activation, and promote postoperative new bone formation. Chien et al. (2018) applied an injectable thermosensitive chitosan/collagen/glycerophosphate hydrogel to provide a suitable environment for the transplantation of stem cells and enhance their delivery and implantation. In an animal model of maxillary molar defects, the iPSCs-BMP-6 hydrogel-treated group exhibited significant mineralization, increased bone volume, number and thickness of bone trabeculae, and promoted new periodontalligament regeneration as well as the formation of bone and cementum. These findings indicate that the combination of hydrogel-encapsulated iPSCs with BMP-6 provides a new strategy for enhancing periodontal bone regeneration.

### 6.2 Hydrogels act as scaffold to promote bone defect repair

In recent years, bone tissue engineering (BTE) has played an important role in the treatment of periodontal bone defects (Gao et al., 2024b). The three important elements in BTE include scaffold materials, seed cells, and growth factors. BTE requires the integration of scaffold materials with different types of bioactive substances, including cells, drugs, proteins, and other bioactive molecules, to enhance bone formation effectiveness. Hydrogels, as scaffold materials, provide a living environment for bone cells, which is conducive to bone tissue regeneration (Peng et al., 2023). Hydrogels can simulate the extracellular matrix environment of the human body, providing structural support for new bone formation and enabling bone tissue repair, thus possessing unique advantages. Sowmya et al. (2017) achieved complete regeneration of hard tissues (alveolar bone and tooth bone) at the site of periodontal defect using a three-layered nanocomposite hydrogel scaffold loaded with growth factors. The results demonstrated complete healing of the periodontal bone defect and the formation of new trabecular-like tissue. Histological and immunohistochemical analysis further confirmed the formation of new tooth bone and alveolar bone, with distinct bone trabeculae. Wang et al. (2023) prepared a porous hydrogel scaffold using chitosan and oxidized chondroitin sulfate (OCS) as the matrix, which carried periodontal ligament stem cells (PDLSCs) or gingival mesenchymal stem cells (GMSCs). In a rat model of periodontal defect, the PDLSC and GMSC hydrogels induced bone tissue repair, offering another possibility for clinical application. Kato et al. (2015) investigated the application of bone morphogenetic protein (BMP)/collagen hydrogel scaffold implantation for periodontal bone defect in dogs. The results showed that bone-like tissue was significantly formed after receiving BMP/collagen hydrogel scaffold, and the hydrogel enhanced the regeneration of tooth bone and alveolar bone.

## 7 Conclusion

The hydrogel is a three-dimensional polymer network characterized by both rigidity and flexibility. Being a rapidly advancing functional polymer material, it possesses distinct advantages that traditional materials lack. It shows significant potential for application in periodontal tissue regeneration and repairing bone defects. Subsequent research should concentrate on enhancing the performance of hydrogels, identifying types with superior performance, and delving deeper into their mechanisms of action. With advancements in medicine, technology, and ongoing research, the utilization of hydrogels in periodontal tissue regeneration and bone defect repair is anticipated to expand new possibilities.
